# Epidemiology and clinical characteristics of patients with healthcare-acquired multidrug-resistant Gram-negative bacilli: a retrospective study from a tertiary care hospital

**DOI:** 10.1038/s41598-024-53596-x

**Published:** 2024-02-06

**Authors:** Banan M. Aiesh, Mustafa Natsheh, Mohammad Amar, Shatha AbuTaha, Mohammad Qadi, Adham AbuTaha, Ali Sabateen, Sa’ed H. Zyoud

**Affiliations:** 1 Infection Control Department, An-Najah National University Hospital, Nablus, 44839 Palestine; 2 Department of Medicine, College of Medicine and Health Sciences, An-Najah National University, Nablus, 44839 Palestine; 3 Department of Biomedical Sciences, Faculty of Medicine and Health Sciences, An-Najah National University, Nablus, 44839 Palestine; 4 Department of Pathology, An-Najah National University Hospital, Nablus, 44839 Palestine; 5 Department of Clinical and Community Pharmacy, College of Medicine and Health Sciences, An-Najah National University, Nablus, 44839 Palestine; 6 Poison Control and Drug Information Center (PCDIC), College of Medicine and Health Sciences, An-Najah National University, Nablus, 44839 Palestine; 7 Clinical Research Center, An-Najah National University Hospital, Nablus, 44839 Palestine

**Keywords:** Microbiology, Diseases, Health care, Medical research

## Abstract

The numbers of infections caused by Gram-negative bacteria (GNB) that produce extended-spectrum beta-lactamases (ESBLs) and those that are carbapenem resistant, especially *Escherichia coli* (*E. coli*) and *Klebsiella pneumoniae (K. pneumoniae*), are increasing, and these infections are becoming a global public health problem. The aim of this study was to assess the prevalence of infections caused by ESBL-producing and carbapenem-resistant Gram-negative bacilli in patients hospitalized at An-Najah National University Hospital in Nablus, Palestine, and to provide healthcare workers with valuable information on the treatment of these infections. A retrospective cross-sectional investigation was conducted at a large tertiary care teaching hospital. The study included patients admitted to the hospital between January and December 2021, from whom ESBL-producing and carbapenem-resistant Gram-negative bacilli were isolated. The patients' clinical and demographic information was obtained from the hospital information system. In addition, information regarding the bacterial isolates and antibiotic resistance was obtained from the hospital's microbiology laboratory. This study included a total of 188 patients—91 males (48.4%) and 97 females (51.6%). The general surgical ward accounted for the highest proportion of infections (30.9%), followed by the surgical ICU (12.2%). The most common infections were caused by ESBL-producing *E. coli,* which accounted for 62.8% of the cases. Among them, urinary tract infections caused by this microorganism were the most prevalent (44.7% of patients). Over 50% of the patients (54.2%) had a history of antibiotic use, and 77.8% had been hospitalized within the past three months. ESBL-producing *E. coli* was significantly isolated from blood cultures (*p*-value = 0.000), and CR-*K. pneumoniae* was significantly isolated from endotracheal isolates (*p*-value = 0.001). This study emphasizes the concerning frequency of healthcare-acquired infections caused by ESBL-producing and carbapenem-resistant GNB in a tertiary care hospital. The substantial prevalence of antibiotic resistance presents considerable obstacles to the successful administration of routinely employed antibiotics. The results highlight the immediate need for improved antimicrobial stewardship and the implementation of infection control strategies to reduce the effects of multidrug-resistant GNB on patient well-being and public health.

## Introduction

The global issue of pathogenic organisms developing antibiotic resistance has grave implications for treating infectious diseases^1,2^. This phenomenon is primarily caused by the excessive or improper use of antibiotics in human medicine, agriculture, and veterinary medicine^3,4^. There is an alarming increase in antibiotic resistance among community- and hospital-acquired pathogens^5,6^.

Continuous exposure of bacterial isolates to a variety of β-lactam antibiotics has resulted in the constant production and mutation of β-lactamases, rendering these bacteria resistant to newly developed β-lactam antibiotics; thus, they are called extended-spectrum β-lactamase (ESBL)-producing pathogens^7^. Treatment of infections induced by these multidrug-resistant organisms is a major concern among scientists^8^. There has been a global increase in ESBL-related infections in recent years^9^. Globally, Gram-negative bacteria are predominantly resistant to β-lactam antibiotics, which are the most common therapeutic option for treating bacterial infections. ESBL-producing Gram-negative bacilli (GNB) can colonize patients without causing acute infection, suggesting that the bacteria are present in certain body sites, such as the nasal and anal cavities, without causing any symptoms^10^. When the immune system is compromised, these organisms become active and cause infection, accompanied by symptoms such as fever, congestion and painful urination. *Escherichia coli* and *Klebsiella pneumoniae* are the most common ESBL-producing pathogens^11^.

*E. coli* and *K. pneumoniae* are among the most common pathogens that cause urinary tract infections. However, *K. pneumoniae* is more strongly associated with catheter-associated urinary tract infections, while *E. coli* is more strongly associated with urogenital problems such as urinary tract stones, neurogenic bladder, or obstructive uropathy^12^.

In 1983, strains that produce ESBL were first reported in Germany. Nonetheless, developing countries have a higher prevalence and thus greater impact of infections caused by bacteria that produce ESBLs. This could be due to certain factors that increase the risk of the emergence of these strains, such as overcrowded hospitals, increased self-medication, and greater usage of nonprescription antimicrobial drugs, which are more common in low-income countries^13–15^.

Infections from ESBL-producing GNB have been increasing dramatically since a study in Sweden showed that the prevalence of ESBL-producing GNB increased from 1.9% in 2008 to 5% in 2010 in their study population. Another 5-year study between 2005 and 2009 demonstrated that the prevalence increased from 5.2% in 2005 to 13.5% in 2009^15,16^. A Centers for Disease Control and Prevention (CDC) report in 2019 showed a 50% increase in hospital- and community-acquired infections of ESBL-producing GNB between 2012 and 2017^17^.

ESBL producers are often resistant to various categories of antibiotics, such as fluoroquinolones and trimethoprim-sulfamethoxazole (TMP/SMX), in addition to the various agents among the β-lactam classes of antibiotics. Although the use of carbapenems is the preferred treatment for severe infections caused by ESBL-producing organisms, there have been reports of carbapenem-resistant infections^18,19^.

Carbapenem-resistant Enterobacterales (CRE) produce carbapenemase and are resistant to meropenem, ertapenem, and imipenem^20^. The first report of CRE was in the early 1990s, and since then, CRE has been disseminated globally^21^. According to the CDC, CREs are recognized as an ‘urgent threat’ to public health since the mortality rate of hospitalized patients infected with CRE is up to 50%^22^. In addition, a study concluded that CRE-infected patients are three times more likely to die than infected patients due to carbapenem-susceptible organisms^23^. Gram-negative bacteria that produce ESBLs and display carbapenem resistance significantly impact patient outcomes, including illness severity and mortality, and are therefore a global healthcare concern. These microorganisms exhibit decreased antibiotic susceptibility, posing treatment challenges. According to a review of 163 studies from low- and middle-income countries that studied antibiotic resistance in hospital-acquired ESKAPE-E *(Enterococcus faecium, Staphylococcus aureus, Klebsiella pneumoniae, Acinetobacter baumannii, Pseudomonas aeruginosa, Enterobacter* spp.*, and Escherichia coli)* infections, the pooled resistance percentage of hospital-acquired ESKAPE-E infections to essential antibiotics ranged from 16.6 to 85.5% and is generally considered high. Additionally, in this systematic review, the proportions of carbapenem-resistant Gram-negative microorganisms were high: 16.6% in *E. coli* and 39% in *K. pneumoniae*. The resistance to third-generation cephalosporins was even greater, as 77% of *K. pneumoniae* and 75% of *E. coli* were resistant to this group of antibiotics^24^.

The aim of this study was to assess the prevalence of infections caused by ESBL-producing and carbapenem-resistant Gram-negative bacilli in patients hospitalized at An-Najah National University Hospital in Nablus, Palestine, and to provide healthcare workers with valuable information on the treatment of these infections. This study is extremely important because multidrug-resistant Gram-negative pathogens are highly drug resistant and pose a threat to public health.

## Methods

### Study design and setting

A retrospective cross-sectional study was conducted from January to December 2021 at Najah National University Hospital (NNUH), Nablus, to investigate the prevalence of infections caused by ESBL-producing Gram-negative bacteria in patients treated for various diseases. NNUH is a nonprofit academic and tertiary care hospital. The hospital contains 135 beds distributed in different wards and departments (surgical, medical, pediatric, and cardiac departments, in addition to their corresponding intensive care units (ICUs), bone marrow transplant, vascular surgery, and emergency departments). The hospital is considered the main cancer center in the northern governorates of Palestine, where patients from all over the West Bank and Gaza Strip are referred to the NNUH.

### Study population

The study included all patients admitted to the NNUH between January 2021 and December 2021 from whom carbapenem-resistant and ESBL-producing *E. coli* and *K. pneumoniae* were isolated. All patients who developed hospital-acquired infections (which occurred 48 h after admission) caused by these pathogens were included in the study. We excluded patients who had these pathogens on admission or within 48 h of admission.

### Data collection

The hospital information system was used to obtain demographic and medical data, while the hospital's microbiology laboratory provided information on the source of the specimen, the type of microorganism, and the antibiotic susceptibility.

### Microbiological methods

Identification and susceptibility testing were performed per the Clinical and Laboratory Standards Institute guidelines using the automated VITEK® 2 Compact system (bio-Mérieux). Identification was performed using VITEK®2 GN ID cards. AST-N204 cards were used to test for antimicrobial agents against aerobic Gram-negative bacilli, including amoxicillin/clavulanic acid, piperacillin, piperacillin-tazobactam, amikacin, imipenem, meropenem, ertapenem, ceftazidime, gentamycin, nitrofurantoin, cefepime, cefotaxime, sulfamethoxazole–trimethoprim, ampicillin and ciprofloxacin^25,26^. Extended-spectrum β-lactamase production was determined using the ESBL test impeded by the AST-N204 card.

### Statistical analysis

The data were entered and analyzed using the Statistical Package for Social Sciences (IBM-SPSS) software, version 21. Continuous variables are displayed as the mean ± standard deviation (SD) or median (interquartile range), while categorical variables are expressed as frequencies and percentages. For categorical variables, the analysis employed either the chi-square test or Fisher’s exact test, as appropriate. All the statistical assessments were two-tailed, with statistical significance defined as a p value less than 0.05, unless explicitly stated otherwise. The reported p values were rounded to three decimal places.

### Ethics approval and consent to participate

The *Institutional Review Board (IRB) of An-Najah National University* approved all aspects of the study protocol, including access to and utilization of patient clinical information. The data collected were exclusively used for clinical research, kept confidential, and not utilized for any other purpose. The project staff had limited access to the collected data, and patient information and hospital names were coded with numbers to ensure anonymity. Since retrospective data were used, the IRB waived the need for informed consent. The study methods adhered to applicable guidelines and regulations.

## Results

### Demographics and clinical profile of the study population

A total of 188 patients were included in our study. Of these, 91 (48.4%) were male, and 97 (51.6%) were female. The patients' median age (IQR) was 53 (32–66).

Nearly half of the patients had a urinary catheter inserted before the infection (50.9%). Thirty-one patients (18.3%) had a central line, and 49 (28.5%) were intubated for more than 48 h before revealing the presence of the pathogen. Approximately 77.8% of the patients had previous hospitalization within the last three months, and 54.2% had a history of prior antibiotic use within the last three months. Among those with comorbid illnesses, 47.1% had malignancies, and 28% had diabetes mellitus. Hypertensive patients composed 35.3% of the patients, 8.8% were on hemodialysis, and 85 patients (49.4%) were on immunosuppressants. The details can be found in Table 1.

**Table 1:** Demographic and clinical profile of patients with infections caused by ESBL-producing and carbapenem-resistant Gram-negative bacilli.

Variable	N (%)
**Age (years), median and IQR**	53 (32–66)
Sex	
Male	91 (48.4)
Female	97 (51.6)
Malignancy	
Yes	81 (47.1)
No	91 (52.9)
Intubation	
Yes	49 (28.5)
No	123 (71.5)
Central line	
Yes	31 (18.3)
No	138 (81.7)
Foley’s catheter	
Yes	87 (50.9)
No	84 (49.1)
Prior antibiotic use	
Yes	91 (54.2)
No	77 (45.8)
Prior hospitalization within the past 3 months	
Yes	133 (77.8)
No	38 (22.2)
Hemodialysis	
Yes	15 (8.8)
No	155 (91.2)
Diabetes mellitus	
Yes	53 (28.1)
No	135 (71.9)
Hypertension	
Yes	61 (35.3)
No	112 (64.7
Immunosuppressed	
Yes	85 (49.4)
No	87 (50.6)
Colonization among those who were screened	
Yes	25 (13.3)
No	163 (86.7)
Total	188

### Hospital wards where infections caused by ESBL-producing or CR Gram-negative bacilli occurred in patients

There were 188 patients with infections caused by ESBL-producing or CR bacteria scattered across different hospital wards. The general surgical ward had the highest number of infections (58; 30.9%), followed by the surgical ICU (23; 12.2%). Numerous infections were also reported in patients treated in the internal medicine department (9.6%), the vascular surgery department (9.0%), the medical ICU (8.0%), the cardiac care unit (CCU) (3.7%) and the pediatric ward (2.7%) (Table 2).

**Table 2:** Locations of infected patients.

Ward	N (%)
General surgery	58 (30.9)
Surgical intensive care unit	23 (12.2)
Unknown	20 (10.6)
Internal medicine	18 (9.6)
Vascular surgery	17 (9)
Medical intensive care unit	15 (8)
Cardiac care unit	7 (3.7)
Pediatrics	5 (2.7)
Pediatric care unit	3 (1.6)
Bone marrow transplant	3 (1.6)
Urology	1 (0.5)
Oncology clinic	18 (9,6)
Total	188 (100)

### Sources of bacteria isolated to produce ESBLs or that exhibit resistance to carbapenems

The greatest number of ESBL-producing or carbapenem-resistant Gram-negative bacilli were isolated from urine (44.7%), followed by from wounds (21.3%), the bloodstream (7.4%), tissues (6.9%), fluids (6.4%) and sputum samples (5.9%), as shown in Table 3.

**Table 3:** Sources of Gram-negative bacilli producing ESBLs or showing resistance to carbapenems.

Source	N (%)
Urine	84 (44.7)
Wound	40 (21.3)
Blood	14 (7.4)
Tissue	13 (6.9)
Fluid	12 (6.4)
Trap	11 (5.9)
Rectal	8 (4.3)
Sputum	3 (1.6)
Bile	2 (1.2)
Missing	1 (0.5)
Total	188 (100)

ESBL-producing *E. coli* was most frequently isolated from patients (62.8%), while the percentage of ESBL-producing *K. pneumoniae* was 18.6%. Resistance to carbapenems was found in 11.2% of *K. pneumoniae* and 7.4% of *E. coli*, as illustrated in Table 4.

**Table 4:** The production of ESBLs and carbapenem resistance among *E. coli* and *K. pneumoniae.*

Species with resistance mechanism	N (%)
ESBL-producing *E. coli*	118 (62.8)
ESBL-producing *K. pneumoniae*	35 (18.6)
*K. pneumonia* CR	21 (11.2)
*E. col*i CR	14 (7.4)
Total	188 (100)

### Antibiotic resistance of ESBL-producing and CR-GNB strains isolated during the study period

Most ESBL-producing Gram-negative bacilli were resistant to ampicillin, cefotaxime, ceftazidime, and cefepime. Among the β-lactamase inhibitor-containing antibiotics, 50.4% of the ESBL-producing *E. coli* were resistant to amoxicillin/clavulanate, while 20.3% were resistant to piperacillin/tazobactam. On the other hand, 64.7% of the ESBL-producing *K. pneumoniae* isolates were resistant to amoxicillin/clavulanate, whereas 28.6% were resistant to piperacillin/tazobactam. In contrast to ESBL-producing *E. coli,* for which only 8.1% were resistant to nitrofurantoin, ESBL-producing *K. pneumoniae* was completely resistant to nitrofurantoin (100%). For gentamicin and amikacin, CR-*K. pneumoniae* was more resistant (61.9% and 71.4%, respectively) than was CR-*E. coli* (57.1% and 14.3%, respectively). *E. coli* and *K. pneumoniae* strains that were resistant to carbapenems also exhibited high resistance to ciprofloxacin (100% and 90.5%, respectively). For ESBL-producing and carbapenem-resistant strains, resistance to trimethoprim/sulfamethoxazole ranged from 74.3% to 78.6%. All these details are shown in Table 5.

**Table 5:** Antibiotic resistance of *E. coli* and *K. pneumoniae strains* producing ESBLs or showing resistance to carbapenems.

Pathogen (n)	Ampicillin	Amoxicillin-clavulanate	Piperacillin-tazobactam	Cefotaxime	Ceftazidime	Ceftriaxone	Cefepime	Ertapenem	Imipenem	Meropenem	Amikacin	Gentamicin	Ciprofloxacin	Nitrofurantoin	Fosfomycin	Norfloxacin	TMP/SMX
Resistant isolates (% of tested)																	
*E. coli* ESBL (118)	118 (100)	59 (50.4)	24 (20.3)	114 (96.6)	114 (96.6)	114 (96.6)	110 (93.2)	0	0	0	2 (1.7)	40 (54.2)	87 (74.4)	5 (8.1)	2 (4.3)	48 (77.4)	87 (75.7)
*K. pneumoniae* ESBL (35)	35 (100)	22 (64.7)	10 (28.6)	35 (100)	34 (97.1)	35 (100)	32 (91.4)	0	0	0	0	15 (42.9)	20 (57.1)	11 (100)	0	4 (36.4)	26 (74.3)
*E. coli* CR (21)	14 (100)	13 (92.9)	13 (92.9)	14 (100)	14 (100)	14 (100)	14 (100)	13 (92,9)	12 (85.7)	13 (92.9)	2 (14.3)	8 (57.1)	14 (100)	3 (50)	1 (20)	6 (100)	11 (78.6)
*K. pneumoniae* CR (14)	21 (100)	21 (100)	21 (100)	21 (100)	21 (100)	21 (100)	19 (90.5)	20 (95.2)	20 (95.2)	20 (95.2)	15 (71.4)	13 (61.9)	19 (90.5)	6 (85.7)	5 (62.5)	7 (87.5)	16 (76.2)

### Antibiotics administered empirically to treat infections caused by ESBL-producing isolates or carbapenem-resistant isolates

The vast majority of empirical antibiotic therapies are used in combination regimens. Piperacillin-tazobactam was the most commonly used antibiotic in 21.8% of the patients, followed by amikacin in 18.4%. The empirical use of ceftriaxone and ceftazidime was observed in 13.8% and 9.6%, respectively, of patients. Colistin was used empirically in 6.4% of patients, and ciprofloxacin was administered in 9.6% of patients, as shown in Table 6.

**Table 6:** Antibiotics used in empiric therapy during the study period.

Antibiotic	N (% of patients)
Piperacillin-Tazobactam	41 (21.8)
Amikacin	34 (18.1)
Vancomycin	32 (17)
Meropenem	26 (13.8)
Ceftriaxone	26 (13.8)
Ceftazidime	18 (9.6)
Ciprofloxacin	18 (9.6)
Metronidazole	17 (9)
Colistin	12 (6.4)
Gentamicin	11 (5.9)
Cefuroxime	11 (5.9)
Levofloxacin	9 (4.8)
TMP/SMX	9 (4.8)
Tigecycline	7 (3.7)
Cefazolin	1 (0.5)
Ampicillin	1 (0.5)
Total	188 (100)

### The presence in different clinical materials of E. coli and K. pneumoniae producing ESBLs or showing resistance to carbapenems

Analysing various clinical materials as a source of resistant isolates, it was found that ESBL-producing *E. coli* were isolated from blood significantly more frequent (*p* = 0.000). Whereas CR-*K. pneumoniae* was isolated significantly more frequent from endotracheal aspirates (*p* = 0.001). Details can be found in Table 7.

**Table 7:** Sources of *E. coli* and *K. pneumoniae* producing ESBLs or showing resistance to carbapenems.

	Total (n)	*E. coli* CR n = 14 N (%)	*K. pneumoniae* CR n = 21 N (%)	*K. pneumoniae* ESBL n = 118 N (%)	*E. coli* ESBL n = 35 N (%)	*P* value
Bile	2	0 (0.0)	0 (0.0)	1 (0.8)	1 (2.9)	0.683
Urine	84	6 (42.9)	7 (33.3)	61 (51.7)	10 (28.6)	0.068
Wound	41	4 (28.6)	2 (9.5)	29 (24.6)	6 (19.7)	0.360
Fluid	12	0 (0.0)	2 (9.5)	9 (7.6)	1 (2.9)	0.506
Sputum	3	0 (0.0)	1 (4.8)	1 (0.8)	1 (2.9)	0.504
Endotracheal aspirate	11	2 (14.3)	5 (23.8)	1 (0.8)	3 (8.6)	< 0.001*
Tissue	13	0 (0.0)	2 (9.5)	6 (5.1)	5 (14.3)	0.185
Blood	14	2 (14.3)	1 (4.8)	3 (2.5)	8 (22.9)	0.001*
Rectal	8	1 (7.1)	1 (4.8)	6 (5.1)	0 (0.0)	0.849

*Significant differences are marked with an asterisk.

## Discussion

Infections caused by drug-resistant GNB, mainly ESBL-producing and carbapenem-resistant pathogens, are becoming a major health threat and their number is increasing dramatically worldwide. These are especially infections caused by *E. coli* and *K. pneumoniaee*^16,27^. Additionally, antibiotic resistance is becoming a global concern due to the overuse and misuse of antibiotics and the lack of antibiotic stewardship programs in hospitals. In addition to increasing morbidity and mortality, infections with resistant organisms lead to increased length of hospital stay^28–30^.

A total of 188 patients were included in our study, 51.6% were female patients and the median age of patients was 53 years. This was similar to other studies that showed female predominance of 65% to 35% in males regarding ESBL- producing *E. coli* infection^31^, similar to another study that also showed female predominance (57%), and the median age was 70 years^32^. Another study in Pennsylvania, USA, revealed the predominance of male gender with 51.5% and a median age of 54^33^.

An Indian study showed that ESBL- producing *E.coli* were most commonly found in urine (40%) followed by pus samples in 22% of cases^31^, which is consistent with our study and other studies which found that urine was a major source of bacterial isolates that produce ESBLs^33,34^. The most statistically significant source of CR *K. pneumnieae* was the sputum of patients intubated endotracheally (endotracheal aspirates), which is confirmed by another study^35^

In our study, the surgical ward and surgical ICU had more cases of Gram-negative bacilli producing ESBLs or showing resistance to carbapenems than the medical ward and medical ICU, which is consistent with another study in New Delhi that showed that 55.8% of infections caused by ESBL-producing *E. coli* were in the surgical ward compared to only 28.6% in the medical ward. In the same study, the similar results were found for ESBL- producing *K. pneumonia*^31^. A study in Saudi Arabia also showed that organisms producing ESBL were most commonly found in the surgical ward^36^ in contrast to that the ICU units had the highest proportion (79%) of isolates in another study in New Delhi^37^.

One of the important results of our study was that approximately half of the patients had prior antibiotic exposure before the culture result showing growth of *E. coli* and *K. pneumonia* producing ESBLs or showing resistance to carbapenems. This was consistent with many studies that concluded that previous antibiotic use, mainly third-generation cephalosporins, increases the risk of developing ESBL- producing GNB^16,38–41^. For instance, a study in South Korea showed that antibiotic exposure within 30 days increases the risk of ESBL-producing GNB infection^42^. This is consistent with our study showing that 54.2% of the patients had previous antibiotic exposure.

Prior or simultaneous colonization with resistant organisms such as ESBL—producing bacteria was a significant risk factor for the development of future infection in many studies^16^. A French study found prior colonization was the main risk factor for subsequent infections^43^. In our study, 13% of the screened patients were found to be colonized with ESBL –producing GNB before the onset of infection. This highlights that routine screening for colonization with ESBL –producing GNB may have clinical implications; it may allow the identification of patients at the highest risk of ESBL—producing GNB infections and facilitate the correct allocation of empiric treatment regimens^44,45^.

We found that approximately 77% of our cases with ESBL –producing GNB were hospitalized within the last three months, in alignment with many other studies showing an association between prior hospitalization and possible ESBL –producing GNB infection^41,46,47^.

A study conducted in India revealed that 58% of ESBL-producing GNBs had a Foley catheter inserted^31^. This finding is similar to that of our study, in which 50.9% of our patients had a Foley catheter inserted. This finding was also similar to that of another study in Pennsylvania, in which 55% of the patients had a urinary catheter inserted^33^. Although 18.3% of our patients had a central line, other studies showed different results; one of those studies showed that only 1.6% of patients had a central venous catheter inserted^31^. In contrast, another study showed that 58% of HELLP syndrome patients had a central venous catheter inserted^33^ and that patients with ESBL-producing *K. pneumoniae* bacteremia were more likely to have invasive devices (urinary and vascular catheters)^48^. In addition, 43% of the patients developed infection 48 h after central venous catheter insertion in a study performed in Missouri in 2018^49^.

Regarding comorbidities, our study showed that 28% of the patients had diabetes mellitus (DM), which is consistent with the findings of a study conducted in Missouri in 2018 in which 29.4% of the 177 patients had DM^49^ and 36% had DM in another study^33^. Similarly, a retrospective study in Missouri showed that 6.8% of patients had end-stage renal disease (ESRD), 15% had end-stage liver disease (ESLD), 19.8% had hematological malignancy, and 20.6% had solid organ malignancy^49^, which is similar and consistent with the findings of our study; malignancy was found in 47.1% of patients, and ESRD was found in 8.8% of patients. Other studies have shown that 12% of cases are malignant^32,40^.

Antibiotic use should be based on local antimicrobial surveillance data and should differ according to the severity of infection, type of infection, source of infection and comorbidities of the patients^43^. A study in Thailand showed that all ESBL-producing *K. pneumoniae strains* (100%) are resistant to ceftazidime and ampicillin and 16.8% are resistant to amikacin^50^. Another study demonstrated high resistance to cephalosporins, fluoroquinolones, and penicillin^31^. These results are similar to those of a previous study conducted in the hematology department of our hospital, which showed 100% resistance to cephalosporins and ampicillin, 75% resistance to levofloxacin, and 62% resistance to TMP-SMX^51^. Another study in India showed similar results; among the *E. coli* isolates, 94% were susceptible in vitro to ertapenem, 96% to imipenem, and 76% to piperacillin-tazobactam. The corresponding percentages for ESBL-producing *K. pneumoniae* isolates were 80%, 84%, and 59%, respectively^52^. These results are similar to our study, which showed high resistance to cephalosporins and less resistance to carbapenems and amikacin. Additionally, all the ESBL-producing isolates in a Palestinian study had MDR patterns and were resistant to the majority of the β-lactam and non-β-lactam antimicrobial agents. Except for carbapenem, these isolates demonstrated minimal susceptibility to the majority of antimicrobial drugs examined. Carbapenem susceptibility has been associated with β-lactam resistance due to ESBL production; therefore, carbapenems are the drug of choice for infections caused by ESBL-producing pathogens^53^. The resistance rate to gentamicin in ESBL-producing pathogens isolated from urine culture ranged from 42.9% to 54.2% in our study, whereas it was 81.8% in another Palestinian study^54^. With regard to imipenem resistance, all the ESBL-producing pathogens in our study were sensitive to this agent, in contrast to the 20% resistance rate to imipenem among ESBL-producing isolates in a Palestinian study conducted by Tayh et al*.*^55^. This might be because patients in hospitals with a high resistance rate are frequently given carbapenems, which may have a role in developing multidrug-resistant bacteria. Carbapenem resistance can be attributed to various mechanisms, such as outer membrane proteins (OMPs) or the production of carbapenemases, extended-spectrum β-lactamases and AmpC plasmid enzymes^56^. Nonetheless, this institution lacks the molecular testing machines needed to explore the molecular mechanism for carbapenem resistance in clinical *Enterobacteriaceae* isolates, for which we were not able to report the exact resistant strains.

Antibiotic stewardship programmes (ASPs) are necessary to minimize the growing threat of antibiotic resistance. Antibiotic stewardship programmes have shown a decrease in the irrational use of antibiotics, thus decreasing the emergence of multidrug-resistant organisms such as ESBL-producing and CR pathogens. This finding is consistent with a study showing a positive impact of ASP on decreasing the incidence of multidrug-resistant organisms, in which the application of ASP led to a decrease in the incidence of ESBL-producing *E. coli* and led to a decrease in the irrational use of ciprofloxacin and cephalosporins^57^.

This research represents a pioneering effort in Palestine to assess the prevalence of infections caused by ESBL-producing and carbapenem-resistant Gram-negative bacilli in patients hospitalized at An-Najah National University Hospital in Nablus, Palestine. The study aims to furnish healthcare professionals with crucial insights into the effective management of such infections. However, our study has several limitations. First, this was a descriptive retrospective study in a single-center hospital; therefore, data for species other than *E. coli or K. pneumnieae* were missing. Third, we did not assess the change in antibiotic resistance throughout the year or annually thereafter.

## Conclusions

Our retrospective analysis provides insight into the frequency and clinical features of healthcare-acquired infections caused by multidrug-resistant GNB, with a specific focus on strains that produce ESBLs and are resistant to carbapenem. This investigation revealed a significant increase in the occurrence of ESBL-producing GNB infections at our institution, reflecting the patterns observed worldwide. The demographic and clinical characteristics of the afflicted patients highlighted the susceptibility of those who had previously been hospitalized, had comorbidities, or a history of antibiotic use. Significantly, the preponderance of infections transpired in the general surgery ward, underscoring the importance of implementing infection control protocols throughout diverse hospital departments. Microbiological research revealed that ESBL-producing *E. coli* were the most prevalent, with urinary tract infections being the primary source of isolation. The reported resistance patterns, especially the elevated rates of resistance to frequently employed antibiotics, provide a significant problem in the empirical treatment of these illnesses. The increase in carbapenem-resistant bacteria adds complexity to treatment choices, as carbapenem resistance is acknowledged as a substantial public health concern by the CDC. However, further research on the risk factors, causes, associations, and antibiotic susceptibility is needed to determine how to reduce the incidence, mortality and morbidity of this disease.

## Data Availability

The data from our surveillance are not available in the public domain due to privacy and ethical restrictions, but anyone interested in using the data for scientific purposes is free to request permission from the corresponding authors.

## Abbreviations

CCU: Cardiac care unit
CDC: Centers for Disease Control and Prevention
CR: Carbapenem-resistant
CRE: Carbapenem-resistant *Enterobacteriaceae*
DM: Diabetes mellitus
ESBL: Extended-spectrum β-lactamase
ESLD: End-stage liver disease
ESRD: End-stage renal disease
GNB: Gram-negative bacteria
HIS: Hospital information system
ICU: Intensive care unit
IRB: Institutional Review Board
NNUH: An-Najah National University Hospital
SICU: Surgical intensive care unit
TMP/SMX: Trimethoprim-sulfamethoxazole
